# Polymer Microchannel and Micromold Surface Polishing for Rapid, Low-Quantity Polydimethylsiloxane and Thermoplastic Microfluidic Device Fabrication

**DOI:** 10.3390/polym12112574

**Published:** 2020-11-02

**Authors:** Chia-Wen Tsao, Zheng-Kun Wu

**Affiliations:** Department of Mechanical Engineering, National Central University, Taoyuan City 32001, Taiwan; qwe19931007@gmail.com

**Keywords:** polymer microfabrication, polymer microfluidics, microchannel, micromold, micromilling, polymer polishing, PDMS casting, thermoplastic hot embossing

## Abstract

Polymer-based micromolding has been proposed as an alternative to SU-8 micromolding for microfluidic chip fabrication. However, surface defects such as milling marks may result in rough microchannels and micromolds, limiting microfluidic device performance. Therefore, we use chemical and mechanical methods for polishing polymer microchannels and micromolds. In addition, we evaluated their performance in terms of removing the machining (milling) marks on polymer microchannel and micromold surfaces. For chemical polishing, we use solvent evaporation to polish the sample surfaces. For mechanical polishing, wool felt polishing bits with an abrasive agent were employed to polish the sample surfaces. Chemical polishing reduced surface roughness from 0.38 μm (0 min, after milling) to 0.13 μm after 6 min of evaporation time. Mechanical polishing reduced surface roughness from 0.38 to 0.165 μm (optimal pressing length: 0.3 mm). As polishing causes abrasion, we evaluated sample geometry loss after polishing. Mechanically and chemically polished micromolds had optimal micromold distortion percentages of 1.01% ± 0.76% and 1.10% ± 0.80%, respectively. Compared to chemical polishing, mechanical polishing could better maintain the geometric integrity since it is locally polished by computer numerical control (CNC) miller. Using these surface polishing methods with optimized parameters, polymer micromolds and microchannels can be rapidly produced for polydimethylsiloxane (PDMS) casting and thermoplastic hot embossing. In addition, low-quantity (15 times) polymer microchannel replication is demonstrated in this paper.

## 1. Introduction

Microfluidics is a highly integrated and multidisciplinary applied science that has been developing for over 30 years [1,2]. Microfluidics has been widely applied in various fields such as public health [3], biology [2,3,4], energy [4,5], chemical engineering [6,7], and environmental science [8,9]. Microfluidic devices are fabricated using numerous materials and approaches. In the early stages of development, microfluidic devices, derived from microelectromechanical systems (MEMSs), were mainly fabricated using silicon microfabrication techniques [10]. However, optical transmissivity is a fundamental challenge that limits optical observation or detection on silicon-based microfluidic platforms. Glass substrates, which have excellent optical performance, are suitable materials for microfluidic device fabrication [11]. However, glass microchannels are mainly generated through photolithography, followed by hydrofluoric acid etching, which is a hazardous process, and the isotropic etching behavior constrains researchers in the construction of various microchannel geometries. Around the 2000s, polymer materials with favorable mechanical, optical, and chemical properties were introduced for the construction of microfluidic systems. Polydimethylsiloxane (PDMS) elastomers [12,13] and thermoplastics such as polymethyl methacrylate (PMMA) and cyclic olefin copolymer (COC) [14,15] are widely used as substrate materials in microfluidics due to their simple and low cost advantages. Other materials such as paper [16] and wood [17] have recently been employed in microfluidics. Among all such materials, polymer materials are still the major substrates used in microfluidics in this stage.

Polymer microfluidic devices can be fabricated through inexpensive and high-throughput manufacturing approaches. Various conventional plastic production processes have been employed for polymer microfluidic device fabrication. Rapid prototyping methods such as computer numerical control (CNC) milling [18], laser ablation [19], or 3D printing [20] can be used to rapidly produce microfluidic devices for a proof-of-concept study at low quantities. For larger quantity requirements, mass production approaches such as injection molding [21], hot embossing [22], or roller imprinting [23] can be used to create polymer microfluidic devices at a low cost. With these advantages, polymers are favorable disposable substrate materials for the microfluidics field and can meet academic research and commercialization requirements [24].

Micromold is a critical tooling technique for making polymer microfluidic device. For thermoplastic-based microfluidic devices, micromold is used to replicate the microchannel through hot embossing or injection molding. For PDMS-based microfluidics, micromold is an essential component in the soft lithography process [25] for casting PDMS microchannels from the mold. Currently, the micromolds are usually constructed through LIGA (Lithographie, Galvanoformung, Abformung), electroplating, dry/wet etching, pulse microelectroforming, or photolithography to generate silicon or metal micromolds [26,27]. SU-8 micromold is the most commonly used method for creating thermoplastic or PDMS microfluidic devices, as it is relatively straightforward and has lower facility costs than other micromold fabrication methods [28,29]. However, adhesion between SU-8 resins and silicon substrates is a potential issue that limits the micromold’s lifetime. The SU-8 layer may peel off during demolding. Furthermore, SU-8 micromold fabrication still requires a cleanroom and UV lithography facilities, limiting accessibility for researchers. Recently, polymer-based micromold has been proposed as a simple alternative to silicon or SU-8 micromold for microfluidic chip fabrication. Polymer micromolds are fabricated through CNC milling [30], laser ablation [31], or 3D printing [32,33]; these are straightforward methods with low-cost desktop fabrication facilities. Such facilities also require fewer operating skills, enabling broad usage [3,34]. However, surface defects, such as milling marks and those due to overheating, or poor printing resolution resulting from the use of related techniques may result in rough microchannels or micromolds or uneven substrate surfaces, limiting microfluidic device performance. Several approaches have been introduced to smooth the surface after milling. For example, Matellan et al. use acetone vapor treatment to smooth the PMMA surface as a cost-effective rapid prototyping method [35] to generate the microfluidic device; or they use cryogenic cooling to polish the acrylic-based polymer for optical lens application [36]. In addition to these approaches, abrasive polishing tools are commonly used to polish plastic parts in large scale [37,38]. This method can also be applied in microfluidics application, e.g., Szymborski et al. use polishing paste and felt to polish the Teflon bioreactor [39]. Although polishing methods have been reported to smooth the microchannel or polymer surfaces, less emphasis has been placed on their use in polymer replications for microfluidics. Therefore, in this study, we introduced a method for polishing polymer microchannels and micromolds for polymer microfluidics. A desktop CNC micromiller was used to generate microchannels and micromolds for rapid prototyping and replication in polymer microfabrication. We evaluated chemical and mechanical polishing performance for microchannel and micromold surfaces. Finally, we used a polished polymer micromold to imprint thermoplastic microfluidic devices and cast PDMS microfluidic devices to demonstrate that the proposed polishing method is simple, cost-effective, and enables the rapid fabrication of micromolds for related applications.

## 2. Experiment

### 2.1. Materials and Reagent

Polymethylmethacrylate (PMMA, Optical grade, CM-205X) is purchased from Chi-Mei Corporation Co., Ltd. (Tainan, Taiwan). Cyclic olefin copolymer (COC, 8007) is purchased from TOPAS Advanced Polymers manufactures (Frankfurt am Main, Germany). Cyclic block copolymer (CBC 010) is purchased from USI Corporation Co., Ltd. (Kaohsiung, Taiwan). Polydimethylsiloxane (PDMS, SYLGARD™ 184) is purchased from Dow Corning Inc. (Atlanta, GA, USA). End mills with 200 µm diameter are purchased from Taiwan Microdrill Co., Ltd. (New Taipei, Taiwan). Wool felt bits (bullet type) are purchased from Dayuan Hardware Co., Ltd. (Taoyuan, Taiwan). PolyWatch (plastic polish) is purchased from EVI GmbH (Neuried, Germany). Acetone (ACE, 99.9%, electronic grade) is purchased from Mingyang Chemical Materials Co., Ltd (Taoyuan, Taiwan).

### 2.2. Micromold and Microchannel Fabrication through CNC Milling

**Figure 1:**
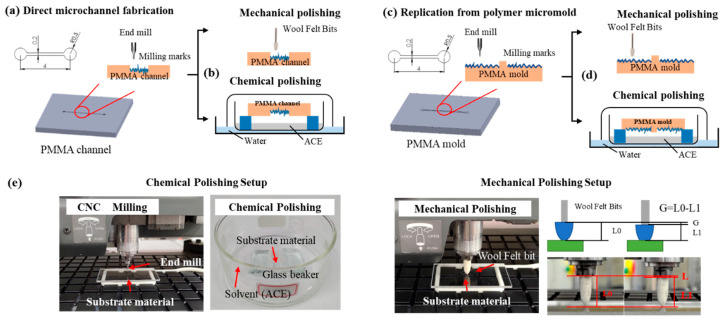
Schematic of (**a**) rapid microchannel prototyping through direct milling for both (**b**) mechanical and chemical polishing. (**c**) shows polymer micromold replication for both (**d**) mechanical and chemical polishing setup. (**e**) displays photographs of the experimental the setup.

As displayed in Figure 1a 6 × 4 mm^2^ PMMA substrate was milled using a CNC micromilling machine (Roland EGX-400, Roland DGA Corporation, CA, USA) to generate a microchannel (Figure 1a) or micromold (Figure 1c) with different spin speeds of 10,000, 20,000, and 30,000 rpm and feed rates of 1, 3, 5, 7, and 9 mm/s. These milling parameters are pre-defined by refereeing related CNC milling investigations [40,41] and pilot trails. We selected polymers with favorable rigidity and thermal properties (i.e., PMMA) as our micromold substrate. The microchannel had a width, length, and height of 200 μm, 4 mm, and 200 μm, respectively, and it had two reservoirs (radius: 500 μm) connected at each end. The micromold surface was subjected to either mechanical or chemical polishing to remove milling marks on the polymer microchannel (Figure 1d) or micromold (Figure 1d) surface. For chemical polishing, we used solvent evaporation to polish the surface, and for mechanical polishing, we used wool felt polishing bits with an abrasive agent (Polywatch) to polish the surface. The polishing process is described in detail in the Discussion section. After polishing, the PMMA substrate was cleaned with deionized water in an ultrasonic cleaner (Delta Ultrasonic Co., Ltd., Delta D150). Figure 1e shows the experimental setup for milling and polishing. For mechanical polishing, we tested different wool felt polishing bit pressing lengths (L). The pressing length is defined as the initial position (Y_0_) subtracted by the polishing position (Y_1_); the related equation is illustrated in Figure 1e.

### 2.3. Surface Roughness and Distortion Percentage Evaluation

For a microchannel and micromold, we evaluated the polymer surface roughness (Ra) by using a surface roughness measuring instrument (SURFCOM 130A, Tokyo Seimitsu Co., Ltd., Tokyo, Japan; sample size: 6 × 6 × 0.2 mm^3^, method: Ra, cutoff (wavelength) λc: 0.8 mm, evaluation length: 3.2 mm, measuring length: 4 mm) and a microscope after both CNC milling and surface polishing. As polishing is either an abrasive or dissolution process, we also evaluated the microchannel and micromold geometry loss after surface polishing. The geometry distortion percentage is defined in Equation (1). Using an inverted fluorescence microscope (Eclipse Ti-U, Nikon Corporation, Tokyo, Japan), cross-sectional images were captured after razor blade cutting of the PDMS replica’s cast from the microchannel and micromolds; the cross-sectional areas were defined before and after polishing.

(1) $$\mathrm{Distortion}\;\left[\%\right]=1-\frac{\mathrm{Cross}-\sec\mathrm{tional}\;\mathrm{area}\;\mathrm{after}\;\mathrm{polishing}}{\mathrm{Cross}-\sec\mathrm{tional}\;\mathrm{area}\;\mathrm{before}\;\mathrm{polishing}}\;\times \;100\;\%$$

## 3. Results and Discussion

### 3.1. Surface Roughness of Different Thermoplastic Surfaces after Milling

**Figure 2:**
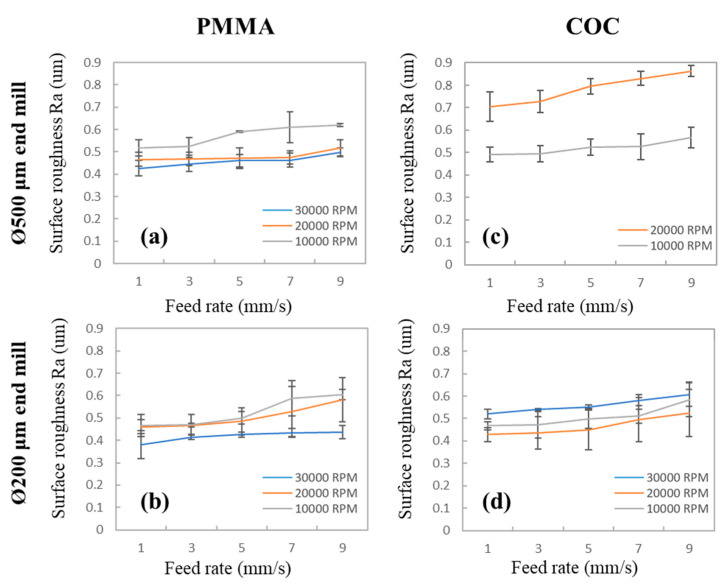
Surface roughness of polymethyl methacrylate (PMMA) (**a**,**b**) and cyclic olefin copolymer (COC) (**c**,**d**) after milling at different feed rates, spin speeds, and end mill diameters. Error bars were obtained based on three individual measurements.

In the fabrication of polymer microfluidic devices using a CNC miller, milling marks that remain on the microchannel surface are a critical concern. PMMA is among the most commonly used thermoplastics in polymer microfluidics due to its low cost and straightforward fabrication. In additional to PMMA, cyclic olefin-based polymers such as COC and cyclic olefin polymer (COP), which have high chemical resistance, are useful for microfluidic applications. Therefore, we first evaluated the surface roughness of PMMA and COC after milling at different spin speeds (10,000 ~30,000 rpm) and feed rates (1~9 mm/s). Both 200- and 500-μm-diameter end mills were selected for fabricating the 200- and 500-μm microchannels. It is noted that although end mills with a minimum diameter of 100 μm are available in the market, we found that end mills with smaller diameters (<100 μm) were easily broken. Thus, to achieve favorable production repeatability and tool lifetime, we chose 200 μm as the minimum end mill diameter in our experiments. The milling test results are displayed in Figure 2. As detailed in the figure, low feed rates improved surface quality; the lowest feed rate of 1 mm/s resulted in optimal surface quality. A high spin speed enables the removal of unwanted materials from the substrate using end mills, resulting in smooth microchannel surfaces. These results agree with those of Chen et al., a high spin speed with a low feed rate reduced surface roughness [40,41]. For PMMA, as shown in Figure 2a,b, lower surface roughness values were found at higher spin speeds, as predicted. However, for the COCs (Figure 2c,d), surface roughness had a weak correlation with spin speed. Machining performance is primarily determined by the polymer’s glass transition temperature (*T*_g_). PMMA, which has a *T*_g_ of 104 °C, has sufficient stiffness for machining. By contrast, in COC substrates, which have a low *T*_g_ (75 °C), due to frictional heat generated during machining at the end mill tip, the COC surfaces are softened or melted at the machining tip. This results in a “wavy” surface with high surface roughness. For the 500-μm end mill, at 30,000 rpm (Figure 2c), we observed that the COC polymers were melt-clogging the end mills, resulting in broken tips. Therefore, the results for the 30,000-rpm experiment are not displayed in Figure 2c.

From the milling tests, we determined the optimum milling conditions for PMMA (Figure 2b—Ø 0.2 mm, 1 mm/s, 30,000 rpm) and COC (Figure 2d—Ø 0.2 mm, 1 mm/s, 20,000 rpm), with minimum surface roughness values of 0.380 μm (PMMA, Figure 2b), 0.428 μm (COC, Figure 2d), respectively. Although using an end mill with a 500-μm diameter generated rougher surfaces, surface roughness only increased by approximately 10%. Considering that the machining time was reduced by approximately 40% and the tool lifetime improved, using a larger end mill (500 μm) is a preferable choice for polymer micromold fabrication. However, in our tests, to determine how to obtain the smoothest surface with minimum dimension loss after polishing, we used 200-μm-diameter end mills for the following tests. Microscope images of the milled PMMA and COC surfaces are shown in Figures S1–S4 in the Supplementary Material. Since PMMA has better thermal stability and a higher *T*_g_ point than COC (104 °C vs. 75 °C), and PMMA is also easy-to-machine polymer, it does not have the end-mill’s tip clogged issues during machining. Thus, for the following polymer microchannel and micromold fabrication, PMMA was used as the major substrate material over COCs. In addition, optimized PMMA processing parameters (Ø 0.2 mm, 1 mm/s, 30,000 rpm) were used to generate an unpolished micromold to investigate polishing performance in the following discussion sections.

### 3.2. Surface Polishing of Microchannels and Micromolds

**Figure 3:**
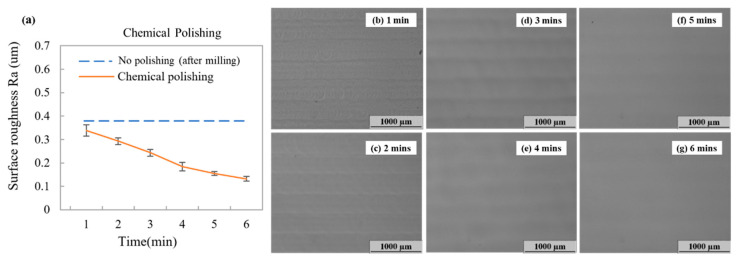
(**a**) Surface roughness of the PMMA surface after chemical polishing with solvent evaporation times of 1, 2, 3, 4, 5, and 6 min. Microscope images of PMMA surfaces at (**b**) 1, (**c**) 2, (**d**) 3, (**e**) 4, (**f**) 5, and (**g**) 6 min. Error bars were obtained based on three individual measurements.

This section provides details of our evaluation of surface roughness and polymer microstructure distortion percentage obtained using chemical and mechanical polishing methods. Both microchannels and micromolds were fabricated, as displayed in Figure 1. Chemical polishing is the most widely used method for polishing related plastic parts. Thus, we first evaluated the effects of chemical polishing on a blank PMMA surface and within the microchannel and micromold substrates. Chemical vapor polishing was performed by fixing the PMMA substrate in an ACE-filled glass beaker, which was heated to vaporize the solvent for polishing the milling marks on the PMMA surface. In our initial trials, we used evaporation temperatures ranging from room temperature (~23 °C) to 80 °C. Excessive surface solvation was observed at a high temperature. Maintaining solvation evaporation at a lower temperature offered better controllability of the polishing process by tuning evaporation times. In a related study, Matellan et al. used acetone to polish a laser-engraved microchannel at 30 °C. They revealed that evaporation at lower temperatures can avoid the generation of visible cracks during polishing [35]. Thus, we chose 40 °C as our solvent temperature and tested different solvent evaporation times (1, 2, 3, 4, 5, and 6 min) to evaluate changes in surface roughness during chemical polishing. As displayed in Figure 3, the surface roughness values of PMMA were reduced as solvent evaporation time increased from 0.338 μm (1 mins) to 0.13 μm (6 min).

**Figure 4:**
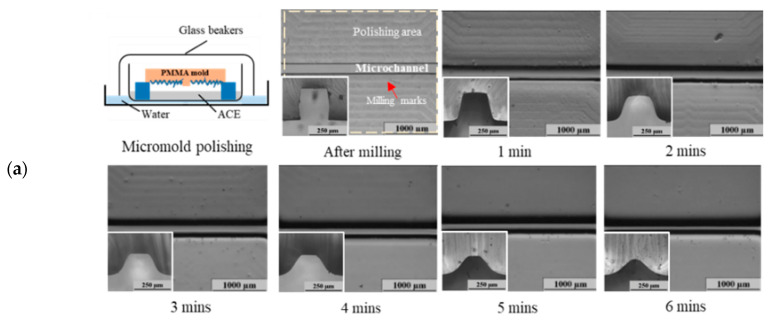

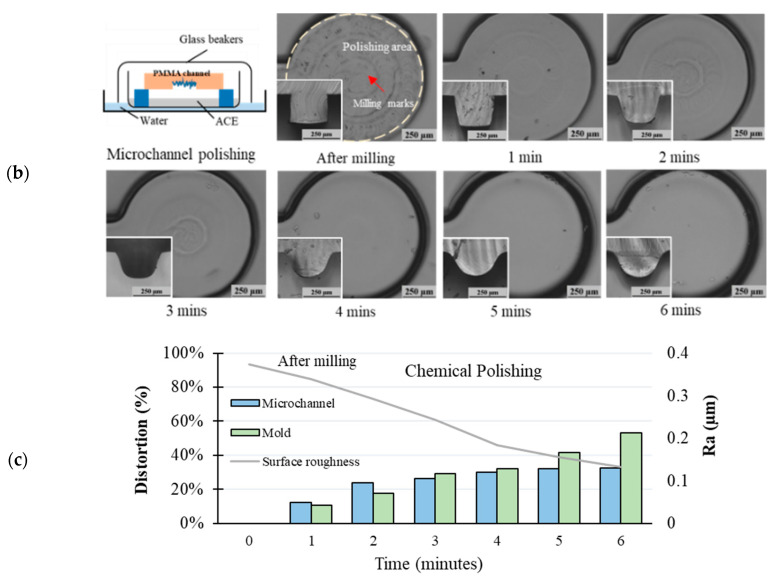
Top and cross-sectional (left-bottom) microscope images showing (**a**) micromolds and (**b**) microchannels immediately after milling (0 min) and after 6 min of chemical polishing. (**c**) Summary of distortion percentage and surface roughness (Ra).

We revealed that chemical polishing is effective at removing milling marks from sample surfaces. Surface roughness was reduced with an increase in the solvent evaporation time. However, for microfluidic applications, the geometric integrity of microstructures must be retained after polishing. Therefore, we further evaluated the microstructural geometric distortions after polishing. Figure 4a,b show the microstructural images of micromolds and microchannels after chemical polishing. As shown in the top and cross-sectional (left-bottom) images in the figure, the original rectangular shape of microstructures changed to hill-like geometries; the hill shapes were more pronounced with a longer polishing time. As summarized in Figure 4c, the distortion percentage from chemical polishing increased from the initial value at 0 min to 53.1% for micromolds and 32.3% for microchannels after 6 min of polishing. These results indicated that although chemical polishing could effectively remove milling marks and irregularities from surface surfaces, microstructure distortion was substantial. From a microchannel fabrication point-of-view, the microchannel should maintain its geometry integrity with original design as close as possible. For microfluidic applications, this factor should be considered when a microchannel’s geometric stability is a crucial issue.

**Figure 5:**
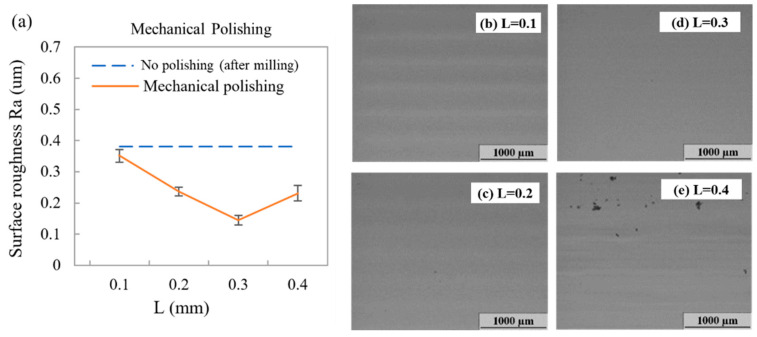
(**a**) Roughness of the PMMA surface after mechanical polishing at pressing lengths of 0.1, 0.2, 0.3, and 0.4 mm. Microscope images of PMMA surface within for pressing lengths of (**b**) 0.1, (**c**) 0.2, (**d**) 0.3, and (**e**) 0.4 mm. Error bars were obtained based on three measurements. For mechanical polishing using a computer numerical control (CNC) machine, the spin speed, feed rate, and overlap path were set as 8000 rpm, 1 mm/s, and 25%, respectively.

Compared with chemical polishing, mechanical polishing using wool felt polishing bits is a more effective and straightforward process; polishing bits are used instead of end mills in an identical CNC machine. The polishing process can be automatically controlled using a CNC machine, with little setup required. For mechanical polishing using wool felt polishing bits, the distance between the wool bit and the PMMA surface (pressing length (L) shown in Figure 1e) is highly correlated with the pressure applied to the polymer surface. Figure 5 shows the surface roughness related to pressing lengths of 0.1, 0.2, 0.3, and 0.4 mm. We defined the original pressing length (L) to be 0 when the wool felt tip touched the PMMA surface. As shown in the figure, surface roughness decreased from 0.380 μm (blue dashed line) to 0.351 μm (L = 0.1 mm) and then to 0.236 μm (L = 0.2 mm). For a pressing length of 0.3 mm, the lowest surface roughness of 0.165 μm was obtained. However, for a pressing length of 0.4 mm, surface roughness increased to 0.231 μm. This is due to wool felt excessively pressing the PMMA surfaces; 5–10-μm dents were observed, indicative of higher surface roughness. For mechanical polishing, an optimal pressing length of 0.3 mm was revealed.

**Figure 6:**
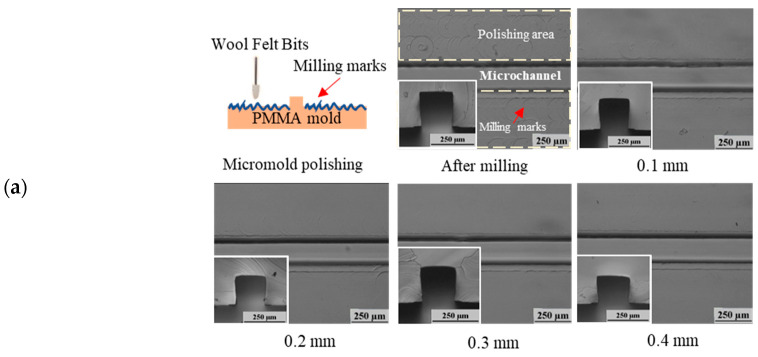

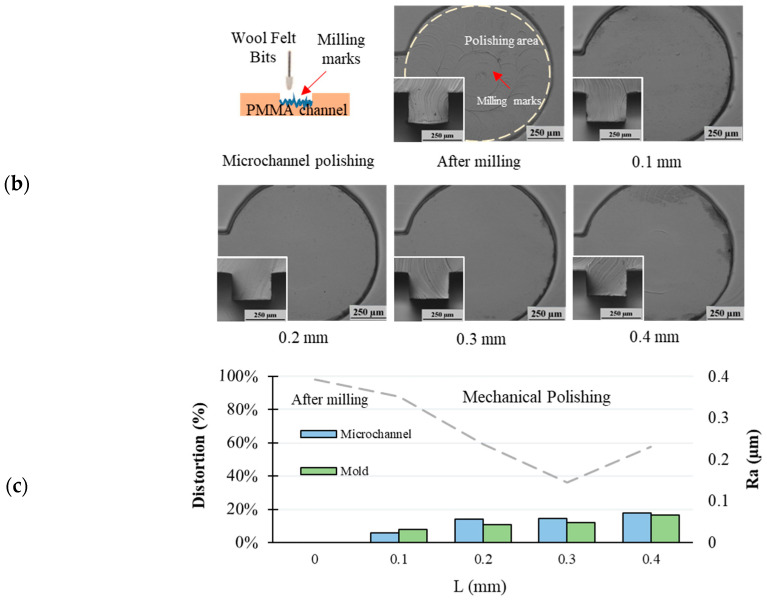
Top and cross-sectional (left-bottom) microscope images of a (**a**) micromold and (**b**) microchannel after milling (0 min) and after mechanical polishing at a pressing length of 0.4 mm. (**c**) Summary of distortion percentage and surface roughness (Ra).

For mechanical polishing, because the wool felt polishing path was controlled by the CNC machine, milling marks were locally polished and removed without causing excessive damage to polymer microstructures. Figure 6 shows top and cross-sectional images of PMMA micromolds (Figure 6a) and microchannels (Figure 6b) after mechanical polishing. The microstructure distortion percentage was only moderately increased for microchannel and micromold fabrication (5.70% and 7.80%, respectively) for a pressing length of 0.1 mm. Wool felt pressing pressure increased with the pressing length. This resulted in lower surface roughness, as discussed previously. This also led to the increased removal of polymer microstructures. In microchannels, for pressing lengths of 0.2, 0.3, and 0.4 mm, the distortion percentage increased to 13.90% (L = 0.2), 14.50% (L = 0.3), and 17.70% (L = 0.4), respectively. In micromold polishing, for pressing lengths of 0.2, 0.3, and 0.4 mm, the distortion percentage increased to 10.80%, 11.80%, and 16.40%, respectively. In mechanical polishing, the highest distortion percentage (53.1%) was obtained. Compared with the chemical approach, mechanical polishing by wool felts was more capable of maintaining the geometric integrity of samples. The cross-sectional images in Figure 6 show that both microchannels and micromolds retained their rectangular-shaped geometries after mechanical polishing.

**Figure 7:**
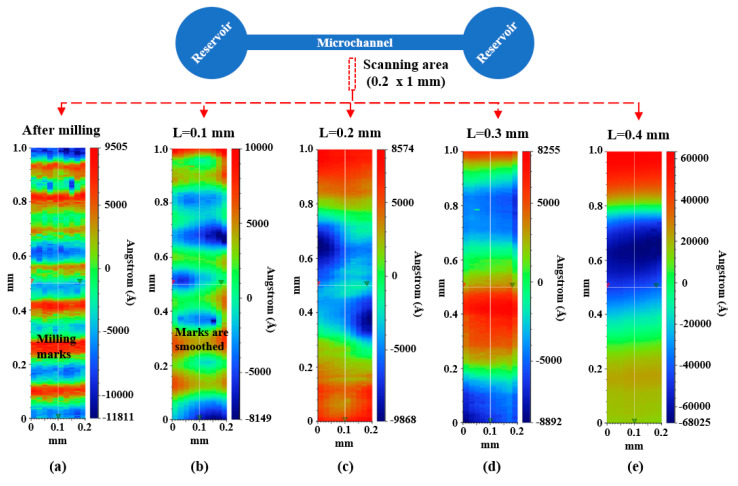
The 3D map of the mechanical polishing micromold analyzed by a surface profilometer. (**a**) After milling and polished with wool felt bit with pressing length (**b**) L = 0.1, (**c**) L = 0.2, (**d**) L = 0.3, and (**e**) L = 0.4.

To further evaluate the surface quality and condition, we performed surface profilometer (DektakXT, Bruker Corporation, Billerica, MA, USA) analysis and collected 0.2 mm × 1 mm surface scanning data for 3D maps of the mechanical polished micromold surfaces as displayed in Figure 7. For the PMMA micromold after CNC milling (Figure 7a), it can be clearly observed that milling marks/defects are uniformly distributed on the PMMA surface. With wool felt bit polishing (L = 0.1 mm), the milling marks were gradually removed (Figure 7b). For increased pressing length (L = 0.2 and 0.3 mm), the milling marks were flattened, generating smoother polymer surfaces (Figure 7c,d) compared to the original condition after milling (Figure 7a). For excessive wool felt bit pressing (L = 0.4), dents may be generated for a rougher surface. Therefore, the moderate pressing length of L = 0.3 is preferred for mechanical polishing.

### 3.3. Thermoplastic and PDMS Microfluidic Device Fabrication Using a Polished Polymer Micromold

**Figure 8:**
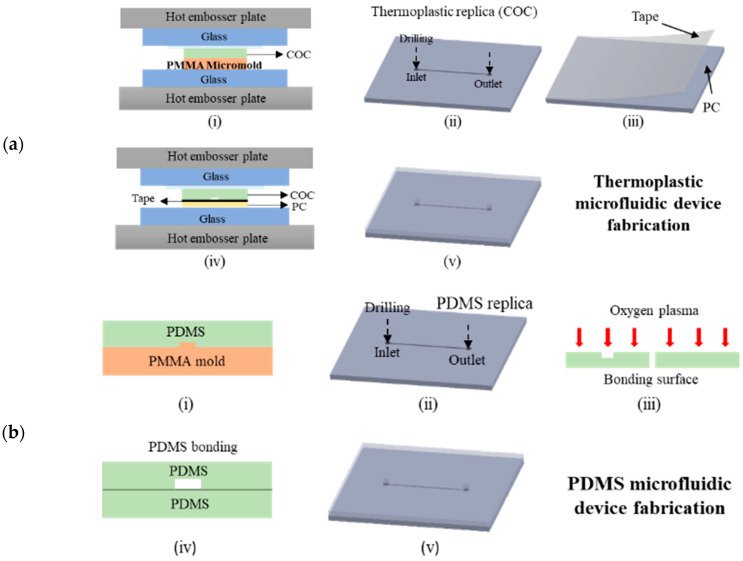
(**a**) Thermoplastic microfluidic device fabrication by hot embossing. (**a-i**) Imprinted COC substrate from a PMMA micromold, (**a-ii**) drill inlet/outlet reservoirs, (**a-iii**) dry adhesive tape applied to the cover substrate (**a-iv**), bond to COC layer, and (**a-v**), completed hot embossing process; (**b**) polydimethylsiloxane (PDMS) microfluidic device obtained by casting. (**b-i**) PDMS from a PMMA micromold (**b-ii**), drill inlet/outlet reservoirs (**b-iii**) oxygen plasma treatment (**b-iv**), bond to PDMS device, and (**b-v**) completed casting process.

Different from SU-8 or other photolithography-based micromolds, polymer-based micromolds fabricated through CNC milling are a bulk material because the PMMA micromold is directly removed from a single substrate. Therefore, no microstructure peel-off occurs, and such micromolds have a longer lifetime than photolithography-based micromolds. However, under excessive heat and pressure application during polymer replication, the microstructures of a polymer micromold may be distorted, which potentially affects the micromold’s lifetime. Thus, we further investigated the geometric stability of micromolds under heat and pressure application during thermoplastic hot embossing and PDMS casting. As shown in Figure 8a, the polished PMMA polymer micromold and COC were sandwiched between glass substrates under a hot embossing machine (Ray Cheng Enterprise Co., Ltd., HT-1). The hot embosser plate was heated to 95 °C at 20 kg/cm^2^ for 3 min to emboss the COC thermoplastic. The hot plate was then cooled to 65 °C, and COC replicas were released from the PMMA micromold. The COC replicas were tape-bonded [42] with another polycarbonate (PC) cover substrate to complete microchannel fabrication. For PDMS microchannel casting, as presented in Figure 8b, PDMS (10:1 PDMS base/curing reagent) was poured onto the PMMA micromold and heated at 60 °C for 5 h. Then, the PDMS replica was released from the PMMA micromold and bonded with another PDMS cover layer using an oxygen plasma process.

**Figure 9:**
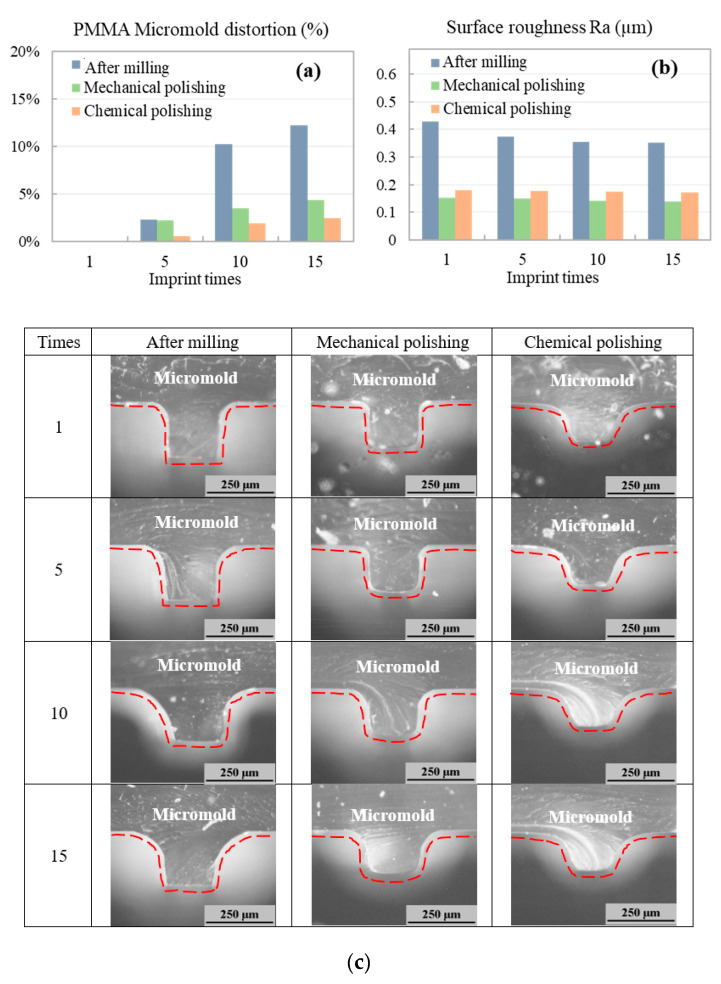
(**a**) PMMA micromold distortion percentage and (**b**) surface roughness after 15 imprint runs. (**c**) Cross-sectional images of micromold surfaces after 1, 5, 10, and 15 imprint runs.

Figure 9 displays the COC microchannel hot embossing results obtained using a polymer micromold. Both optimized mechanically polished (0.3-mm pressing length) and chemically polished (4-min solvent polishing time) polymer micromolds were compared with polymer micromolds after milling (without polish, control sample). We measured the COC microchannel surface roughness and microchannel distortion percentage after imprinting, and 15 microchannel replications (imprint runs) were created to examine micromold production repeatability. Although the hot embossing temperature was lower than the PMMA micromold Tg, the micromold could still distort under heat (95 °C) and pressure (20 kg/cm^2^) application during hot embossing because thermoplastic is a semicrystalline material. As shown in Figure 9a, the shape of the mechanically polished micromold changed (distortion percentage: 4.4%) after 15 imprint runs. The distortion percentage of the chemically polished micromold was distorted 2.4% after 15 imprint runs. For the surface roughness, for the micromold without surface polishing, the roughness reduced from 0.43 to 0.35 μm. With surface polishing micromold, since surface defects were already removed during the polishing step, surface roughness remained almost constant after 15 imprint runs for both mechanically (0.14–0.15 μm) and chemically polished (0.17–0.18 μm) micromolds. Thus, compared with polymer micromolds after milling (without polishing, dark-blue bars in Figure 9), both the mechanically and chemically polished micromolds had better repeatability and smoother surfaces after more polymer imprint runs. This also indicates that residue stress or surface defects on the CNC-milled micromold can effectively be removed by the proposed polishing methods for higher geometric stability.

**Figure 10:**
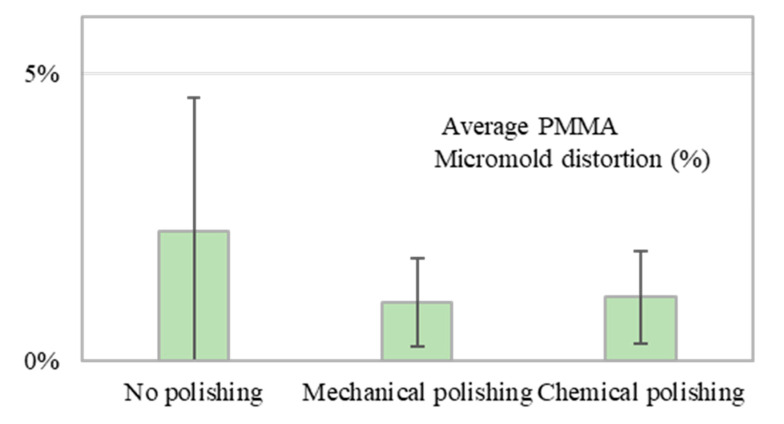
PMMA micromold distortion percentage for PDMS casting in no-polish, mechanical polishing, and chemical polishing conditions. The error bars were obtained based on five individual measurements.

PDMS casting is another widely used method for manufacturing polymer microfluidic devices; it requires the use of micromolds to replicate PDMS microchannels. For PDMS casting, because no pressure is applied in moderate thermal curing (60 °C), polymer micromolds have better repeatability and higher geometrical stability for PDMS casting procedures than other materials. Figure 10 shows the PMMA micromold distortion percentage based on five casting measurements (each individual measurement and corresponding photographs are displayed in Figure S5 of the Supporting Information). Mechanically and chemically polished micromolds had minimum micromold distortion percentages of 1.01% ± 0.76% and 1.10% ± 0.80%, respectively. This demonstrates that polishing PMMA micromolds could improve their stability for casting compared with a no-polish condition (2.33% ± 2.66%).

## 4. Conclusions

Polymer materials with advantages of high performance, low cost, and disposability have been widely applied in the field of polymer microfluidics. CNC milling is a straightforward and effective method for fabricating polymer microfluidic devices. Microchannels can be directly milled for prototyping to rapidly fabricate microchannels or use micromolds to manufacture polymer microchannel replicas in large quantities. However, post milling surface marks may affect microfluidic device performance. In this study, we introduced chemical and mechanical surface polishing approaches for removing surface defects on microchannels and micromolds after milling. The results showed that through chemical polishing, surface roughness values decreased from 0.38 μm (0 min, after milling) to 0.13 μm after 6 min of evaporation time. However, after 6 min of solvent polishing, the distortion percentages of microchannels and micromolds reached 32.3% and 53.1%, respectively. For mechanical polishing, because the polishing area was locally controlled by a CNC router, mechanical polishing using wool felt polishing bits provided favorable surface roughness with smaller geometric loss compared to chemical approach. Surface roughness was reduced to 0.165 μm for the optimal pressing length of 0.3 mm. After wool felt polishing, the microchannel distortion percentage was 14.50%, and micromold distortion percentage was 11.80%, which successfully demonstrates that the mechanical polishing method can be used to polish microchannel and micromold with lower distortion percentage compared to the chemical method.

Finally, we evaluated unpolished, chemically polished, and mechanically polished PMMA micromolds for thermoplastic (COC) hot embossing and PDMS casting replication. The results revealed distortion percentages of 2.4% and 4.4% for chemically and mechanically polished micromolds, respectively, after 15 COC imprint runs. For PDMS casting, because the related materials are subject to less heat and pressure application compared with hot embossing process, the geometric integrity of the PMMA micromold’s structure was maintained (with ~1% variation) after each casting. The results presented herein demonstrate that by using surface polishing methods to satisfy low-quantity requirements, polymer micromolds can be rapidly produced for PDMS casting and thermoplastic hot embossing.

## Supplementary Materials

The following are available online at https://www.mdpi.com/2073-4360/12/11/2574/s1, Figure S1: Microscope images of PMMA surface using Ø500 μm diameter end mills with different spin speeds and feed rates; Figure S2: Microscope images of PMMA surface using Ø200 μm diameter end mills with different spin speeds and feed rates; Figure S3: Microscope images of COC surface using Ø500μm diameter end mills with different spin speeds and feed rates; Figure S4: Microscope images of COC surface using Ø200 μm diameter end mills with different spin speeds and feed rates; Figure S5: Cross-sectional images of PMMA micromold after 5 PDMS casting runs.
