# Supplementary material for: Polymer Microchannel and Micromold Surface Polishing for Rapid, Low-Quantity Polydimethylsiloxane and Thermoplastic Microfluidic Device Fabrication
Source: Polymers (Basel). 2020 Nov 2;12(11):2574. doi: 10.3390/polym12112574 (PMC7692984; doi:10.3390/polym12112574)
Supplement: Supplementary file 1 [file polymers-12-02574-s001.pdf]

Polymer Microchannel and Micromold Surface Polishing for Rapid, Low-Quantity Polydimethylsiloxane and Thermoplastic Microfluidic Device Fabrication

Chia-Wen Tsao* and Zheng-Kun Wu

Department of Mechanical Engineering, National Central University, Taoyuan City, Taiwan

*Corresponding author.
E-mail: cwtsao@ncu.edu.tw
Tel: +886-3-4267343
Fax: +886-3-4254501

Photograph images & surface roughness Ra (μm)		Feed rate (mm/s)				
		1	3	5	7	9
Speed (RPM)	10000	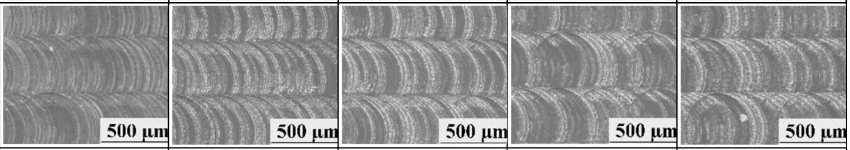				
		0.517μm	0.524μm	0.590μm	0.609μm	0.620μm
	20000	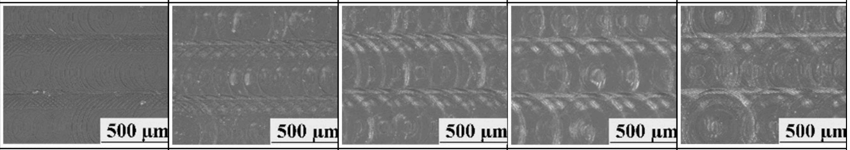				
		0.466μm	0.467μm	0.472μm	0.474μm	0.517μm
	30000	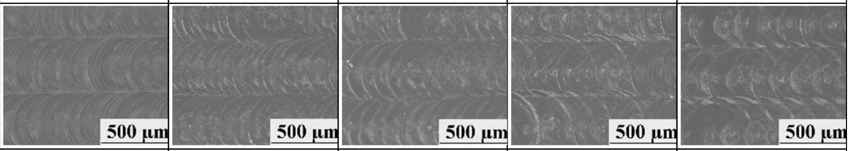				
		0.426μm	0.444μm	0.461μm	0.463μm	0.498μm

Fig. S1 Microscope images of PMMA surface using Ø500 μm diameter end mills with different spin speeds and feed rates.

Photograph images & surface roughness Ra (μm)		Feed rate (mm/s)				
		1	3	5	7	9
Speed (RPM)	10000	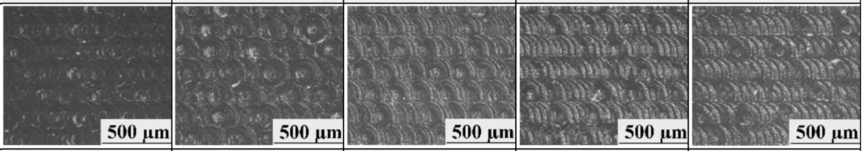				
		0.466μm	0.470μm	0.500μm	0.588μm	0.604μm
	20000	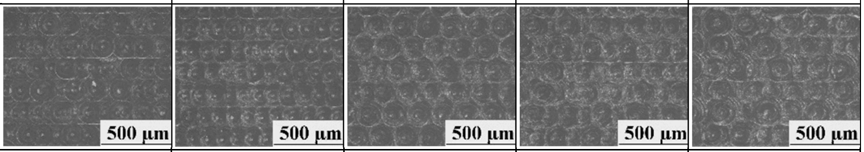				
		0.460μm	0.467μm	0.484μm	0.527μm	0.581μm
	30000	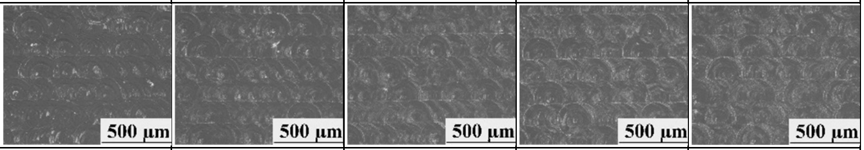				
		0.380μm	0.413μm	0.425μm	0.433μm	0.436μm

Fig. S2 Microscope images of PMMA surface using Ø200μm diameter end mills with different spin speeds and feed rates.

Photograph images & surface roughness Ra (µm)		Feed rate (mm/s)				
		1	3	5	7	9
Speed (RPM)	10000	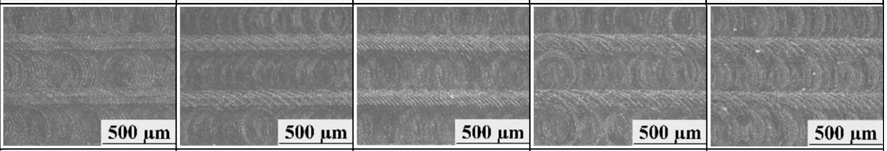				
		0.492µm	0.494µm	0.525µm	0.526µm	0.567µm
	20000	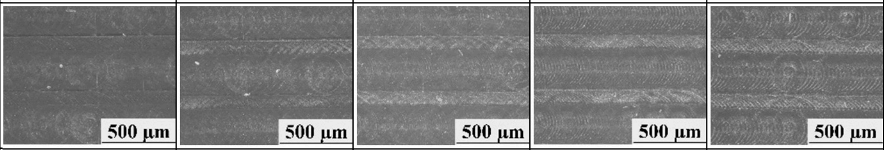				
		0.705µm	0.728µm	0.795µm	0.831µm	0.862µm

Fig. S3 Microscope images of COC surface using Ø500µm diameter end mills with different spin speeds and feed rates.

Photograph images & surface roughness Ra (µm)		Feed rate (mm/s)				
		1	3	5	7	9
Speed (RPM)	10000	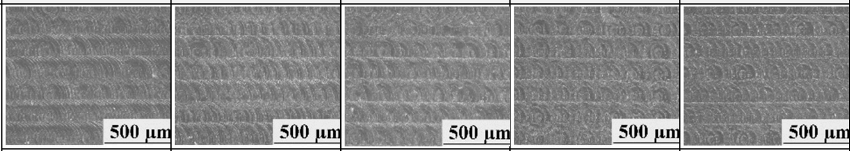				
		0.468µm	0.473µm	0.497µm	0.510µm	0.585µm
	20000	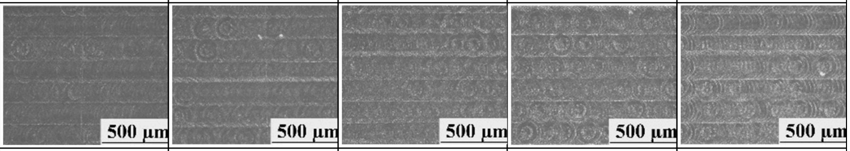				
		0.428µm	0.436µm	0.449µm	0.495µm	0.523µm
	30000	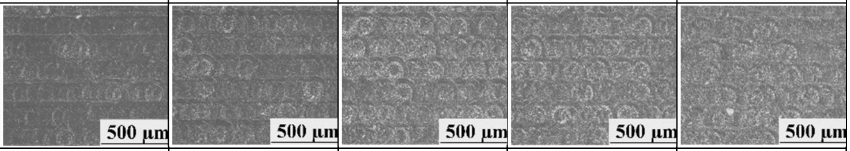				
		0.520µm	0.539µm	0.552µm	0.581µm	0.606µm

Fig. S4 Microscope images of COC surface using Ø200µm diameter end mills with different spin speeds and feed rates.

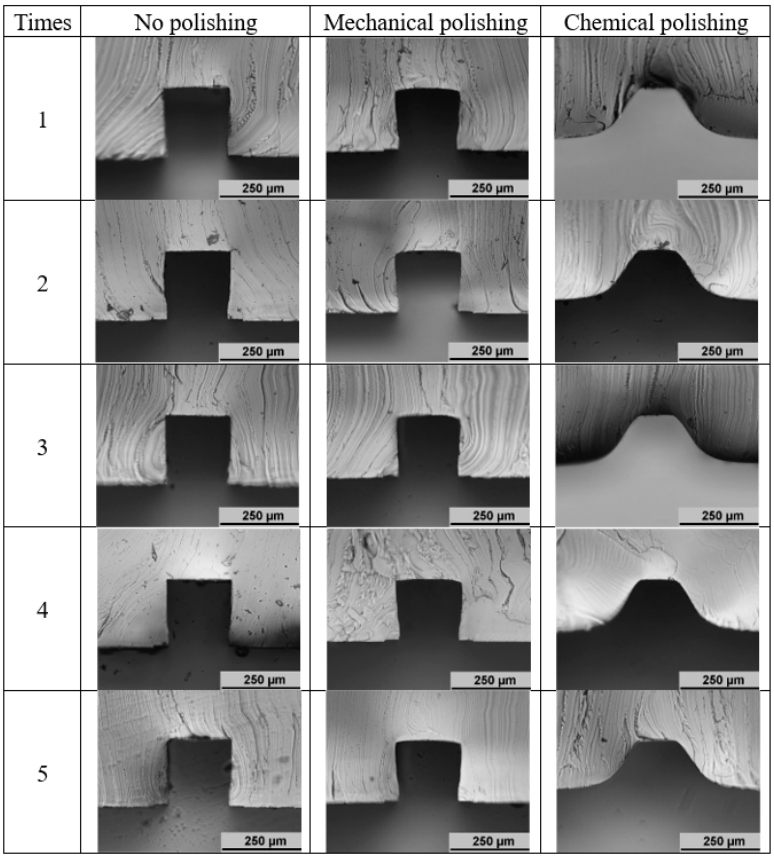

Fig. S5 Cross-sectional images of PMMA micromold after 5 PDMS casting runs
